# Cascara Sagrada in Rheumatism

**Published:** 1888-09

**Authors:** 


### CASCARA SAGRADA IN RHEUMATISM.

Dr. Goodwin, of the United States Marine Hospital Service, has discovered that cascara sagrada possesses remarkable powers for the relief of rheumatism. Encouraged by the results accidentally obtained on his own person, he tried it on a number of patients suffering from well-marked rheumatic manifestations, most of whom had been treated with more or less unsuccess by means of the salicylates, iodides, etc. Within twenty-four hours he was surprised and gratified to find a marked improvement in every case. One particularly striking case was that of a Swedish soldier, who had been suffering intensely for three months, none of the usual remedies affording him more than transient relief. Fifteen-drop doses of the fluid extract of cascara sagrada three times a day produced a marked improvement in forty-eight hours, and by the seventh day he had so far recovered that he received permission to walk out. From that date he steadily improved and made a satisfactory recovery. In some cases it may advantageously be combined with salicylate of sodium. The action on the bowels was seldom excessive, and the laxative effect usually produced was a distinct advantage. Dr. Goodwin confesses himself unable to explain the action of the drug in relieving rheumatism, but this, after all, is not of much importance if only his conclusions bear the test of experience.—*Medical Press and Circular.*

